# Reinforcement Mechanism of Carbon Black-Filled Rubber Nanocomposite as Revealed by Atomic Force Microscopy Nanomechanics

**DOI:** 10.3390/polym13223922

**Published:** 2021-11-12

**Authors:** Xiaobin Liang, Makiko Ito, Ken Nakajima

**Affiliations:** Department of Chemical Science and Engineering, Tokyo Institute of Technology, 2-12-1 Ookayama, Meguro-ku, Tokyo 152-8552, Japan; ito.m.av@m.titech.ac.jp (M.I.); knakaji@mac.titech.ac.jp (K.N.)

**Keywords:** visualization, stress distribution, atomic force microscopy, reinforcement, rubber

## Abstract

In this study, atomic force microscopy (AFM) nanomechanics were used to visualize the nanoscale stress distribution in carbon black (CB)-reinforced isoprene rubber (IR) vulcanizates at different elongations and quantitatively evaluate their volume fractions for the first time. The stress concentrations in the protofibrous structure (stress chains) that formed around the CB filler in CB-reinforced IR vulcanizates were directly observed at the nanoscale. The relationship between the local nanoscale stress distribution and macroscopic tensile properties was revealed based on the microscopic stress distribution and microscopic spatial structure. This study can help us gain insight into the microscopic reinforcement mechanism of carbon black-containing rubber composites.

## 1. Introduction

Mixing nanoscale fillers with a polymer matrix to produce polymer nanocomposites (PNCs) is of substantial and growing importance. PNCs are widely used in many practical applications, and their reinforcement mechanism and structure have been the subject of numerous experimental studies over recent decades [1,2,3,4,5]. In the rubber industry, the incorporation of stiffer nanoscale inclusions significantly improves the elastic modulus, fracture strength, and abrasion resistance of the rubber matrix [6,7]. The stiffening effect largely depends on the type of filler (carbon black (CB) [8], silica [9], layered silicates [10], and clay sheets [11]), volume fraction, and dispersion. The reinforcement of composites is generally believed to result from interactions between the filler and rubber based on the “bound rubber” theory [12,13]. The concept of “bound rubber” is defined as the insoluble rubber phase in an organic solvent due to the adsorption of macromolecular chains to the filler particle surface. Nishi [14] observed that the molecular motion in the layer between CB and rubber was unaffected by the solvent by measuring the spin–spin relaxation T2 time using pulsed nuclear magnetic resonance (NMR), which indicates strong restriction of the molecular motion of rubber on the CB surface. Using low-field proton nuclear magnetic resonance (NMR), Litvinov et al. [15] also observed significantly different ethylene propylene diene monomer (EPDM) rubber chain mobilities in CB-filled EPDM and strong immobilization of EPDM chain fragments on the surface of CB. Valentin et al. [16] studied polymer-filler interfaces by combining swelling experiments with low-field NMR. They found that the interactions established between the polymer chains and the filler restrict the swelling process in the elastic chains near the filler surface and hinder the swelling process in the interface. Additionally, hydrodynamic reinforcement contributes to the stiffening effect because rigid filler particles are incorporated in soft rubbery matrices. Gold, Guth, and Vilgis et al. [17,18] presented a theoretical model of hydrodynamic reinforcement based on the Einstein-Smallwood formula [19] to describe the relationship between elastic modulus and volume fraction of the filler. Recently, Long et al. [20] revealed, using a new microscopic model, that strong reinforcement was achieved when glassy layers among fillers overlapped, and the reinforcement was very strong when the relevant fillers with glassy layers percolated. Using three-dimensional transmission electron microscopy (3D-TEM), Ikeda et al. [21] first visualized nanofiller networking due to the association of CB and natural rubber. Based on the aforementioned descriptions, we found that the reinforcement mechanism of PNCs was complex due to the diversity of fillers and their spatial arrangement, and the microscopic reinforcing mechanism and mechanical behavior of this action remain unresolved.

To investigate the reinforcement mechanism of PNCs, information on the microscopic scale under deformation conditions, i.e., spatial dispersion and interactions between the rubber and fillers, is necessary. The dispersion and relative motions of the fillers upon deformation have been investigated using X-ray [22,23] and neutron diffraction [24,25]. These studies have difficulty directly obtaining information about local structures at the nanoscale and analyzing the diffraction data due to the form factor of the particles. Recently, transmission electronic microscopy (TEM) [26,27] and atomic force microscopy (AFM) [28,29] have been used to investigate local structures upon deformation and filler dispersion in real space. For example, Fretigny [30] used AFM to investigate the dispersion of fillers in nanocomposites under uniaxially stretched conditions. Real space images illustrate that the existence of many-body interactions can lead to remarkable alignments of the fillers perpendicular to the traction direction. Díez-Pascual et al. [31,32] explored the interfacial properties of fibrous fillers and polymeric matrices using a nanoindentation technique. Using TEM, Yatsuyanagi et al. [33] observed the breakdown of the secondary structure of silica particles when strain was applied to silica-filled vulcanizates. Jinnai et al. [34] conducted in situ nanoscale observations on the crack propagation process in silica nanoparticle-filled rubber. These phase imaging techniques provide good contrast between the rubber matrix and the fillers, which enables access to real space data and assists in understanding the reinforcement mechanisms. However, these techniques commonly cannot thoroughly probe the reinforcing mechanism because they only provide information related to the morphology and spatial dispersion of fillers. No techniques can provide information related to stress localization. New methods that can provide clear experimental data for the mechanical modeling of PNCs are necessary.

Recently, AFM nanomechanical measurements have become a straightforward and efficient method for high-spatial-resolution imaging of material properties [35,36,37,38,39]. Previous studies on CB-filled natural rubber (NR) using AFM nanomechanical measurements have focused on the microstructure and elastic modulus of bound rubber and have shown that bound rubber has a two-layer structure consisting of a glassy layer and an uncured rubber layer and that bound rubber exhibits a higher elastic modulus than matrix rubber [40]. In this study, we proposed an approach to measure the stress distribution of CB-reinforced IR at the nanoscale using PeakForce Quantitative NanoMechanics (QNM) mode and a specially designed holder to keep the sample in a uniaxially stretched position. This approach can directly visualize the nanoscale stress distribution of CB/IR at various elongations and quantitatively assess its volume, which enables the acquisition of information about the stress localization and interfacial structure of the filler/rubber. More importantly, this approach enables the application of a micromechanical analytical model to provide insight into the microscopic reinforcement mechanism of CB-filled rubber composites.

## 2. Materials and Methods

### 2.1. Materials and Sample Preparation

The PNCs in this study were a mixture of isoprene rubber and high-abrasion furnace-grade CB (N330, with a mean particle size of 28–36 nm). Its formulations are presented in Table 1. All ingredients were commercially available. The loading contents of CB were 10, 20, 30, 40, and 50 phr, which corresponded to 4.9%, 9.1%, 13.2%, 16.7%, and 20.2% in volume fraction, respectively. A specially designed sample holder was used to maintain the CB/IR sheets (20 mm × 2 mm × 0.2 mm) in a uniaxially stretched condition under the AFM probe. Prior to stretching, two lines normal to the stretching direction were marked on the surface of the samples. The macroscopic strain can be precisely calculated from the measurement of the distance between two lines. Then, the deformed samples were ultramicrotomed using a Leica EM FC6 (Leica Microsystems GmbH Wetzlar, Germany) at −120 °C to obtain a smooth surface for AFM imaging. Cutting was performed parallel to the tensile direction.

### 2.2. AFM Measurements

**Figure 1:**
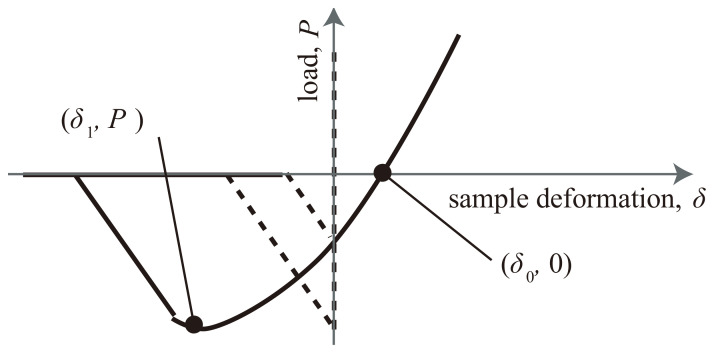
A schematic of the force–deformation curve.

AFM measurements were performed in PeakForce Quantitative NanoMechanics (QNM) mode on a Bruker MultiMode AFM at ambient conditions. The oscillation frequency of the Z-piezo was 1.0 kHz, and the peak-force amplitude was set at 250 nm. Force curves were collected over randomly selected surface areas of 3.0 μm at a resolution of 256 pixels × 256 pixels. The samples were scanned at peak tapping forces of approximately 1 nN using rectangular silicon cantilevers with a nominal spring constant of 0.51 N/m (OMCL-TR800PSA, Olympus Micro Cantilevers). The actual spring constant was measured using the thermal tuning method. The obtained force curves were analyzed using JKR contact mechanics [41]. According to this model, the Young’s modulus *E* and adhesive energy *w* are expressed by two equations:

(1) $$E=\frac{3\left(1-v^{2}\right)}{4}\frac{-1.27P}{\sqrt{R{\left(\delta_{0}-\delta_{1}\right)}^{3}}}$$

(2) $$w=-\frac{2P}{3\pi R}$$

where *ν* is the Poisson’s ratio, *R* is the radius of curvature of the probe tip, *δ* is the sample deformation, and *P* (<0) is the maximum adhesive force, as shown in Figure 1 [42].

### 2.3. Macroscopic Tensile Measurements

Tensile tests were performed using a Shimadzu EZ-TEST instrument under uniaxial tension at a cross-head speed of 50 mm/min at room temperature. During tensile testing, the samples were first stretched to a desired strain at room temperature; then, the strain was maintained for 1 h to stabilize the stress relaxation. We performed such procedures to quantitatively compare the macro- and microscale mechanical properties because the AFM nanomechanical mapping measurements were performed after stretching the samples for 1 h. Dumbbell-shaped mini tensile bars (40 mm × 2 mm × 2 mm) were formed by cutting from 2-mm-thick CB/IR sheets, and at least five measurements were performed for each sample.

### 2.4. Analysis of Nanoscale Stress Distribution

Nanomechanical mapping measurements can be used to analyze the nanoscale stress distribution of deformed material. However, the elastic modulus maps measured in the deformed state cannot directly quantify the stress distribution on the material surface. In general, the modulus under deformation is greater than that of undeformed material, and we suggest that this increase in modulus is directly related to the stress distribution. Therefore, in this study, we approximated the stress distribution as an increase in modulus during deformation. As a result, we define the stress σ as follows:

(3) $$\sigma =E_{Str}-E_{Unstr}$$

where *E_str_* and *E_Unstr_* are the Young’s moduli of deformed and undeformed rubber, respectively. To verify the above analysis method, we compared the modulus increase of polydimethylsiloxane (PDMS) with the macroscopic tensile stress. Both were almost in agreement (see Figure S1 and Table S1), which proves the validity of Equation (3).

## 3. Results and Discussion

**Figure 2:**
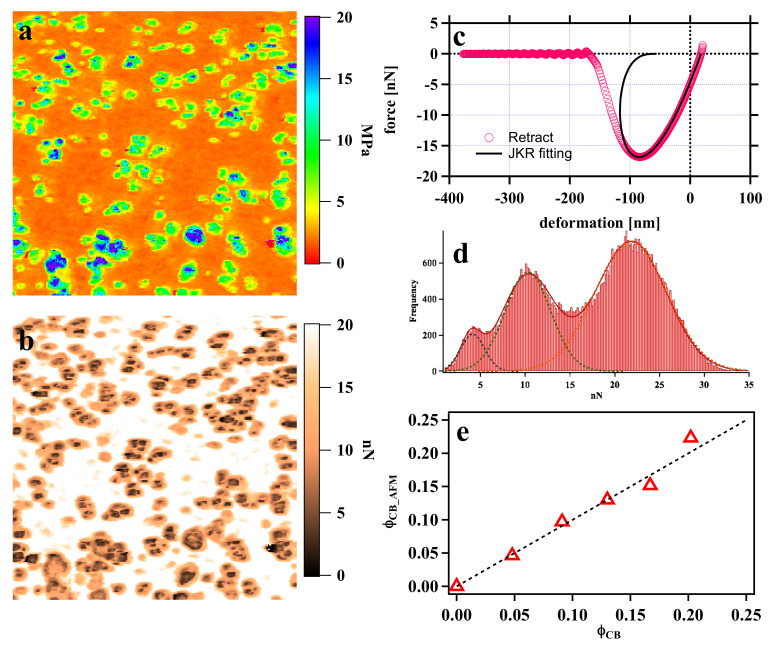
(**a**,**b**) Young’s modulus and adhesion force mapping of CB (4.9%, 10 phr)/IR obtained by PeakForce QNM AFM. The scan size was 3.0 μm; (**c**) typical force−deformation curve. The Young’s modulus results from a fit of the retracing curve with a JKR contact model, as indicated by the solid black line. (**d**) Histograms of adhesion force mapping for CB (4.9%, 10 phr)/IR. (**e**) Volume fraction of CB from PeakForce QNM AFM and the real formulation.

**Figure 3:**
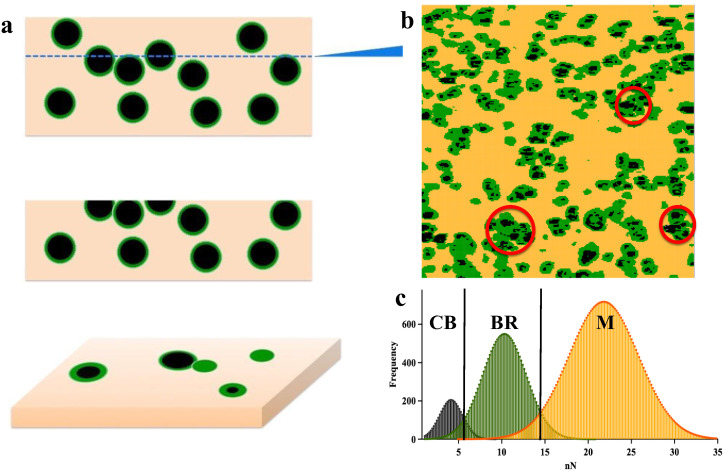
(**a**) Schematic for CB/IR before cutting and after cutting. (**b**) Visualization of the dispersion structure of carbon black in IR by AFM adhesion force images, where the black area is CB, the yellow area is IR rubber, and the green area is the interface. (**c**) Histograms of adhesion force mapping for CB, IR and the interface.

We used the PeakForce QNM mode to measure the local mechanical properties of a material at the nanoscale. The Young’s modulus and adhesion images of the undeformed CB (4.9 vol%)-reinforced IR vulcanizate are provided in Figure 2a,b. The estimated Young’s modulus image shows the interfacial region between the CB with a higher modulus (blue–green) and the IR matrix with a lower modulus (orange). Here, the elastic modulus of CB was ~22 MPa (Figure S2), which is much lower than the typical modulus value of CB (~10 GPa) [43]. The underestimated elastic modulus of CB results because the surrounding rubber region is deformed [44]. Another possibility is that the applied force was too small to deform CB particles because a soft probe was used (spring constant was 0.51 N/m for the rubber-level measurement). In this case, it is very difficult to define the CB region and interfacial region using the difference in modulus. Instead, the histogram of the adhesion force can be well described by the Gaussian function in Figure 2d, which clearly shows three relatively independent Gaussian distributions. The notable difference in surface adhesion energy is caused by different molecular structures of CB, the IR matrix, and the interface between CB and the IR matrix. These three Gaussian distributions correspond to CB, interfaces, and IR matrices from small to large, respectively. By fitting the histogram (Figure 2d), the calculated volume fraction of CB, *ϕ_CB_*, is 4.6%, which is consistent with the original filler content of 4.9 vol%. The comparison between the volume fraction of CB from PeakForce QNM AFM and the real formulation at various *ϕ_C__B_* values showed that they were consistent through the loading content, as shown in Figure 2e. The adhesion force can accurately locate and quantify the CB region in the CB/IR vulcanizate. Adhesion force mapping can be used to probe the local structural information of CB and estimate the volume fraction of the interface and IR matrix with *ϕ_IF_* = 23.9% and *ϕ_M_* = 71.5%. Tensile testing revealed that the effective volume fraction f was approximately 1.8 in the modified Guth-Gold equation [17,18]:

(4) $$E_{f}=E_{m}\left(1+2.5f\phi +14.1f^{2}\phi^{2}\right)$$

where *E_f_* and *E_m_* are the Young’s moduli of the filled composite and rubber matrix, respectively, and *ϕ* is the volume fraction of filler. According to the AFM results, (*ϕ_IF_* + *ϕ_CB_*)/*ϕ_CB_* = 6.1, which indicates that f increases approximately threefold in comparison with the value obtained from tensile testing. Two possible reasons can lead to the increased interfacial region in AFM mapping. First, the CB aggregates under the rubber surface or the tool only cut the interfacial region, as shown in Figure 3a, which leads to an overestimation of the interfacial region. Second, the IR matrix may be confined to a certain area, which results in similar mechanical properties to those of the interfacial region (red circle in Figure 3b).

**Figure 4:**
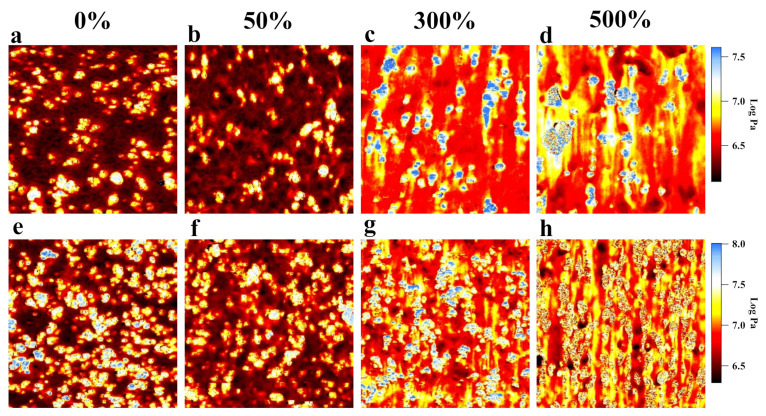
Modular images of CB (4.9%, 10 phr)/IR in 3.0 × 3.0 μm areas: (**a**) unstretched; (**b**) stretched to *λ* = 50%; (**c**) stretched to *λ* = 300%; (**d**) stretched to *λ* = 500%. Modular images of CB (13.2%, 30 phr)/IR in 3.0 × 3.0 μm areas: (**e**) unstretched; (**f**) stretched to *λ* = 50%; (**g**) stretched to *λ* = 300%; (**h**) stretched to *λ* = 500%.

Figure 4 presents the nanomechanical mapping results for CB (4.9 vol%, 10 phr) and CB (13.2 vol%, 30 phr)-reinforced IR at various elongations during uniaxial stretching. The logarithm of the modulus was used in this mapping, which makes it easy to observe structural changes during uniaxial stretching. Based on the results for the definition of the CB, interface, and IR (Figure 3b), the Young’s modulus was 1.9 ± 0.3 MPa for the rubber matrix, 4.0 ± 1.8 MPa for the interface component, and 9.8 ± 0.3 MPa for the CB component (as noted above, the Young’s modulus of CB was untrue; therefore, we will not discuss it in this article). When CB (4.9 vol%, 10 phr)/IR was stretched to *λ* = 50% (Figure 4b), the heterogeneity of the elastic modulus in the IR matrix was revealed by AFM nanomechanical mapping. This result implies that affine deformation cannot be applied to the extension of the CB/IR. When the sample was stretched to *λ* = 300%, the nanomechanical mapping results indicate that the modulus increased to 4.4 ± 1.6 MPa in the IR matrix region and to 8.5 ± 2.7 MPa in the interfacial region. The stress concentration of fibrillar structures (stress chain) formed around CB fillers and oriented along the tensile direction (blue circle area in Figure 4c). The stress tended to be concentrated in the interfacial regions, where the local rubber molecular chain was bound to the CB particles, which led to dramatically decreased molecular mobility. With further deformation (Figure 4d), the stress chain expanded along the stretching direction, and the CB fillers connected with each other to form a network structure. This structure resulted in dramatically increased macroscopic stress at high elongations. Furthermore, the area of the interfacial region significantly increased with stretching at high elongation, which indicates that the stress was again concentrated in the interfacial region. At high volume fractions of CB (Figure 4e–h), a similar process was observed by nanomechanical mapping. For the samples with higher CB volume fractions, chain stress structures of high-density networks were observed, which led to a high elastic modulus and low fracture of the material.

For a long time, various particulate reinforcement theories (such as parallel [45], series [46,47], Hirsch, Chow [48], and Takayanagi [49] models) were proposed to predict the mechanical properties of polymer composites, and all theories have been based on either a micromechanical or phenomenological approach. However, we have long been unable to directly obtain the microscopic mechanical properties of the materials. PeakForce QNM AFM enables the qualitative analysis of the stress distribution of the material surface and provides the possibility of quantitative characterization of the nanostress distribution. A new model can be established to provide insight into the relationship between the local and nanoscale stress distributions and macroscopic tensile properties. For CB-reinforced IR systems, a modulus of 10 GPa for CB is much larger than that of the rubber matrix (a few MPa), which indicates that the CB cannot sustain stress when the sample is stretched. Consequently, the problem has been simplified to a two-phase model, where stresses and strains are considered in the rubber and interface. Here, two basic parallel and series models were discussed and applied to the deformation system for the first time. The parallel model is given by the rule of mixtures:

(5) $$\sigma_{par}=\sigma_{IF}{\phi^{\prime }}_{IF}+\sigma_{M}{\phi^{\prime }}_{M}$$

(6) $${\phi^{\prime }}_{IF}=\phi_{IF}/\left(\phi_{IF}+\phi_{M}\right)$$

(7) $${\phi^{\prime }}_{M}=\phi_{M}/\left(\phi_{IF}+\phi_{M}\right)$$

where *σ_par_* is any mechanical property of the CB-reinforced IR system, *σ_IF_* and *σ_M_* are the microscopic stresses of the interface and matrix components, respectively, and *ϕ_IF_* and *ϕ_M_* are their corresponding volume fractions. In the series model, the blend components are arranged in series, and the equation is given as follows:

(8) $$\sigma_{ser}={\left({\phi^{\prime }}_{IF}/\sigma_{IF}+{\phi^{\prime }}_{M}/\sigma_{M}\right)}^{-1}$$

**Figure 5:**
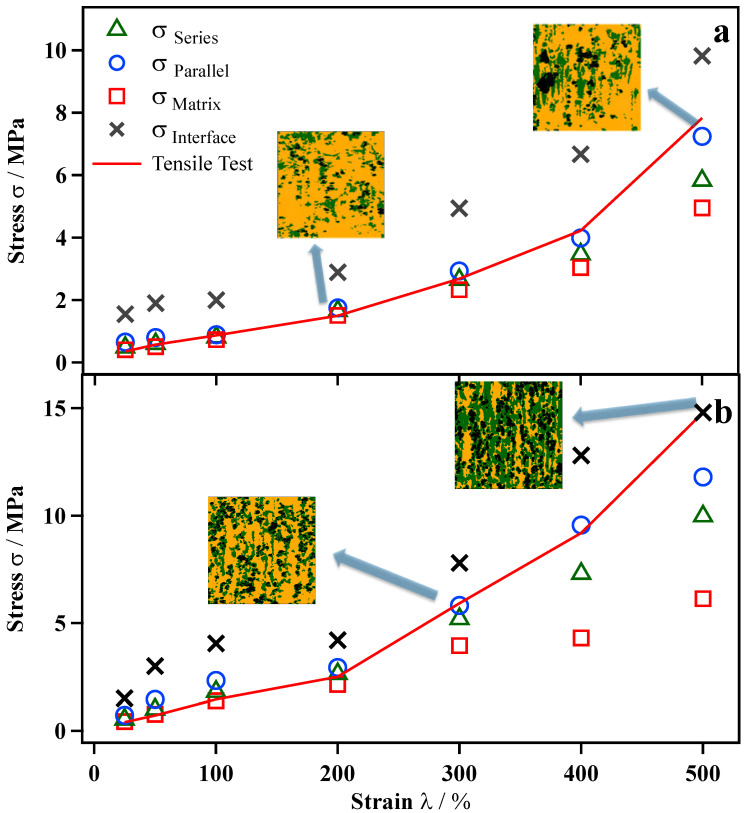
Comparison of macroscopic tensile data (red line) and PeakForce QNM AFM results (point) based on series and parallel model discussed in the text for stress of CB (4.9%, 10 phr)/IR (**a**) and CB (13.2%, 30 phr)/IR (**b**) as a function of the elongation. Because the CB does not actually carry the stress, it is reduced to a two-phase model here. The black area is CB, the yellow area is IR rubber, and the green area is the interface.

As shown in Figure 1, the elastic modulus of nanomechanical mapping includes the material modulus itself and stress concentration under stretching; therefore, part of the stress can be extracted by Equation (3) and is shown in Table S2. Figure 5 shows the comparison of macroscopic tensile data and PeakForce QNM AFM results based on the series and parallel models discussed in the stress of CB/IR as a function of the elongation section. In the case of CB (4.9 vol%, 10 phr)/IR (Figure 5a), the macroscopic tensile stress was consistent with the tensile stress of the IR matrix at elongations of 0%~200%. Furthermore, when the sample was stretched to 300%, the macroscopic tensile stress agreed with the series model, and the series arrangement of the rubber phase and interphase were observed by nanomechanical mapping. After the rubber was stretched to 400%, the tensile stresses were close to the parallel model, which indicates that the rubber and interface form a parallel structure. The macroscopic tensile stress of the stretched CB/IR depends on (i) the microscopic local stress distribution of the interfacial region and IR matrix region and (ii) the microscopic spatial structure of each phase. We first successfully obtained the relationship between the local, nanoscale stress distribution and macroscopic tensile properties. In the case of CB (13.2 vol%, 30 phr)/IR (Figure 5b), a parallel structure appeared at lower elongations. A possible explanation of this phenomenon is that a higher loading content makes forming stress-chain networks easier. Interestingly, when the CB (13.2 vol%, 30 phr)/IR sample was stretched to 500%, the macroscopic tensile stress was consistent with the tensile stress of the interface because the interfacial area increased with the stretch to form a matrix at elongations of 500%, which caused a stress almost distributed in interfacial areas. Therefore, the microscopic stress distribution and microscopic conformations of the interface and matrix together determine the microscopic mechanism of the CB-reinforced IR rubber under deformation.

## 4. Conclusions

In summary, we used AFM nanomechanics to measure the nanoscale mechanical property distribution of CB/IR vulcanizate under uniaxial tension for the first time. Many stress chains formed around CB fillers parallel to the traction direction in stretched CB/IR. Moreover, the stress chains could be connected to form a network structure at high elongations, which resulted in high macroscopic stress. Using the differences in adhesion force between the tip and surface, the boundaries of each component (CB, interface and IR matrix) of CB/IR were defined by nanomechanical mapping, which enabled the quantitative characterization of the nanoscale stress distribution of stretched material for the first time. Analysis of the stress distribution indicated that macroscopic tensile stress could be predicted by the reinforcement model based on the microscopic stress distribution and microscopic spatial structure. This approach can provide new research perspectives for the reinforcement and deformation mechanisms of PNCs.

## Figures and Tables

**Table 1:** Formulation of the CB-reinforced isoprene rubber.

Component	Composition (phr)
isoprene rubber, IR	100
Sulfur	2
Stearic acid	1
Zinc oxide	5
N-cyclohexylbenzothiazole-2-sulfenamide, CBS	1
Carbon black, CB	0, 10, 20, 30, 40, 50

## Supplementary Materials

The following are available online at https://www.mdpi.com/article/10.3390/polym13223922/s1, Figure S1: Comparison of macroscopic tensile stress curves and microscopic stresses for PDMS, Figure S2: (a) Young’s modulus mapping of HAF-CB (4.9 vol%, 10 phr) reinforced IR obtained by PeakForce QNM AFM. (b) Section analysis of Young’s modulus, Table S1: Young’s modulus *E_Unstr_*, *E_str_* of deformed and undeformed rubber for PDMS, and stresses *σ* calculated by Equation (3), Table S2: Local stress distribution of interface and rubber regions for CB/IR of CB (4.9 vol%, 10 phr) and CB (13.2 vol%, 30 phr).
