# Supplementary material for: Reinforcement Mechanism of Carbon Black-Filled Rubber Nanocomposite as Revealed by Atomic Force Microscopy Nanomechanics
Source: Polymers (Basel). 2021 Nov 12;13(22):3922. doi: 10.3390/polym13223922 (PMC8618764; doi:10.3390/polym13223922)
Supplement: Supplementary file 1 [file polymers-13-03922-s001.zip › polymers-1443191-supplementary.pdf]

polymers

MDPI

Article

Reinforcement Mechanism of Carbon Black-Filled Rubber Nanocomposite as Revealed by Atomic Force Microscopy Nanomechanics

Xiaobin Liang *, Makiko Ito and Ken Nakajima

Department of Chemical Science and Engineering, Tokyo Institute of Technology, 2-12-1 Ookayama, Meguro-ku, Tokyo 152-8552, Japan; ito.m.av@m.titech.ac.jp (M.I.); knakaji@mac.titech.ac.jp (K.N.)
* Correspondence: liang.x.ac@m.titech.ac.jp; Tel.: +81-3-5734-2944

Citation: Liang, X.; Ito, M.; Nakajima, K. Reinforcement Mechanism of Carbon Black-Filled Rubber Nanocomposite as Revealed by Atomic Force Microscopy Nanomechanics. *Polymers* **2021**, *13*, 3922. https://doi.org/10.3390/polym13223922

Academic Editor(s): Marcin Masłowski

Received: 15 October 2021
Accepted: 9 November 2021
Published: 12 November 2021

Publisher's Note: MDPI stays neutral with regard to jurisdictional claims in published maps and institutional affiliations.

Copyright: © 2021 by the authors. Licensee MDPI, Basel, Switzerland. This article is an open access article distributed under the terms and conditions of the Creative Commons Attribution (CC BY) license (http://creativecommons.org/licenses/by/4.0/).

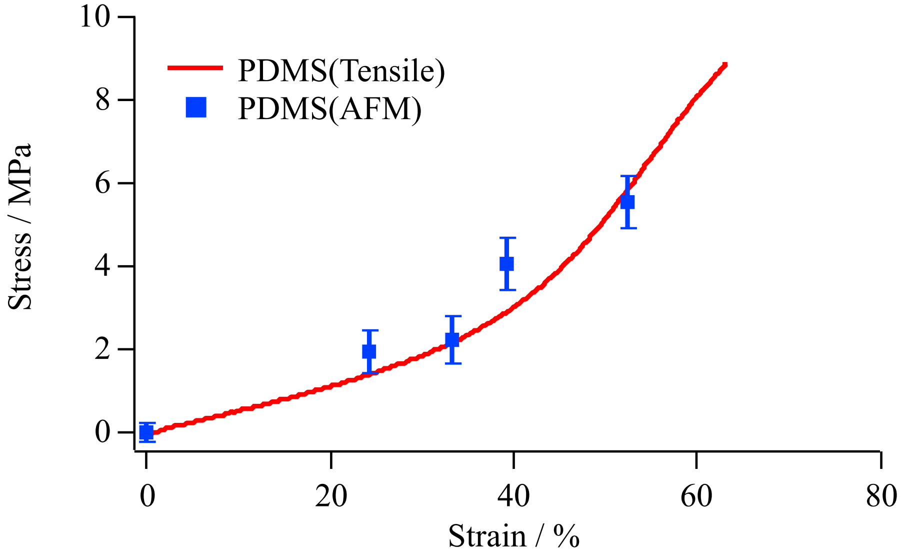

Figure S1. Comparison of macroscopic tensile stress curves and microscopic stresses for PDMS. The two are almost identical, which proves that our analytical model is correct.

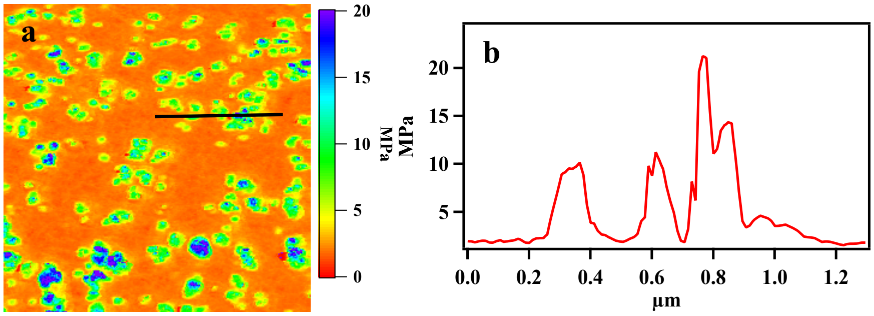

Figure S2. (a) Young's modulus mapping of HAF-CB (4.9%) reinforced IR obtained by PeakForce QNM AFM. (b) Section analysis of Young's modulus.

Table S1. Young's modulus E_{unstr}, E_{str} of deformed and undeformed rubber for PDMS, and stresses σ calculated by Equation 3.

**Strain %**	**0**	**24**	**33**	**39**	**53**
E_{unstr}	3.8 ± 0.2 MPa	-	-	-	-
E_{str}	-	5.7 ± 0.4 MPa	6.0 ± 0.5 MPa	7.9 ± 0.5 MPa	9.4 ± 0.5 MPa
σ	0	1.9 ± 0.5 MPa	2.2 ± 0.6 MPa	4.1 ± 0.6 MPa	5.6 ± 0.6 MPa

Table S2. Local stress distribution of interface and rubber regions for CB/IR of CB (4.9vol%) and CB (13.2vol%).

**Strain %**	$\sigma_{M\text{-}CB(4.9vol\%)}$	$\sigma_{IF\text{-} CB(4.9vol\%)}$	$\sigma_{M\text{-}CB(13.2vol\%)}$	$\sigma_{IF\text{-} CB(13.2vol\%)}$
25	0.4 MPa	1.6 MPa	0.4 MPa	1.5 MPa
50	0.5 MPa	1.9 MPa	0.8 MPa	3.0 MPa
100	0.7 MPa	2.0 MPa	1.4 MPa	4.1 MPa
200	1.6 MPa	2.9 MPa	2.2 MPa	4.2 MPa
300	2.5 MPa	4.9 MPa	4.0 MPa	7.8 MPa
400	3.0 MPa	6.7 MPa	4.3 MPa	12.8 MPa
500	4.9 MPa	9.8 MPa	6.1 MPa	14.8 MPa
